# Signatures of selection in the genome of Swedish warmblood horses selected for sport performance

**DOI:** 10.1186/s12864-019-6079-1

**Published:** 2019-09-18

**Authors:** Michela Ablondi, Åsa Viklund, Gabriella Lindgren, Susanne Eriksson, Sofia Mikko

**Affiliations:** 1 Dept. of Animal Breeding and Genetics, Swedish University of Agricultural Sciences, PO Box 7023, S-750 07 Uppsala, Sweden; 2 Department of Veterinary Science, Università degli Studi di Parma, 43126 Parma, Italy; 3 Livestock Genetics, Department of Biosystems, Leuven, KU Belgium

**Keywords:** Haplotype homozygosity, Horse, Performance, Runs of homozygosity, Selection signature

## Abstract

**Background:**

A growing demand for improved physical skills and mental attitude in modern sport horses has led to strong selection for performance in many warmblood studbooks. The aim of this study was to detect genomic regions with low diversity, and therefore potentially under selection, in Swedish Warmblood horses (SWB) by analysing high-density SNP data. To investigate if such signatures could be the result of selection for equestrian sport performance, we compared our SWB SNP data with those from Exmoor ponies, a horse breed not selected for sport performance traits.

**Results:**

The genomic scan for homozygous regions identified long runs of homozygosity (ROH) shared by more than 85% of the genotyped SWB individuals. Such ROH were located on ECA4, ECA6, ECA7, ECA10 and ECA17. Long ROH were instead distributed evenly across the genome of Exmoor ponies in 77% of the chromosomes. Two population differentiation tests (F_ST_ and XP-EHH) revealed signatures of selection on ECA1, ECA4, and ECA6 in SWB horses.

**Conclusions:**

Genes related to behaviour, physical abilities and fertility, appear to be targets of selection in the SWB breed. This study provides a genome-wide map of selection signatures in SWB horses, and ground for further functional studies to unravel the biological mechanisms behind complex traits in horses.

## Background

The Swedish Warmblood (SWB) is a modern horse breed selected for equestrian sport purposes, mainly show jumping and dressage [1]. The origin of the breed dates back to the eighteenth century when the Royal Cavalry requested more agile and faster horses, leading to intensified breeding and selection of Swedish riding horses [2]. The SWB studbook was thus founded in 1928 with the initial aim to breed horses for multiple equestrian purposes. Since the demand for physical and mental abilities in sport horses has increased noticeably in the last decades, emphasised selection of SWB horses for specific disciplines, which are dressage or show-jumping, is now common practice. The current goal of the SWB studbook is to breed internationally-competitive warmblood horses in terms of rideability, performance-oriented temperament, excellent gaits and/or jumping ability [1].

Recent advances in genomic methodologies have paved the way to explore the effects of selection in the genome. Positive selection reduces genetic variability and it results in increased genomic homozygosity. Stretches of consecutive homozygous loci, runs of homozygosity (ROH), have been used to identify genomic regions potentially under artificial selection in several species. The first large-scale study of how selection has shaped the equine genome involved 744 horses from 33 breeds, genotyped using the Illumina SNP50 Bead chip. The study was based on fixation index (F_st_) statistics and provided evidence of genomic selection signatures for breed-specific morphological features and gaits [3]. Genomic regions with loci responsible for body size, milk yield and fertility were detected by ROH analysis in cattle [4–6], whereas regions involved in immune system and behavioural features were discovered in sheep and goats [7, 8]. More recent examples of ROH and population structure analyses in horses were based on both medium and high-density genotype data and revealed key aspects on the history of European horse breeds [9–12]. ROH detected on whole-genome sequencing data from ten horses of different breeds showed potential selection for reproduction and fertility-related traits [13]. Similarly, the degree of homozygosity at haplotype level can be used to detect signatures of positive selection [14]. The Cross Population Extended Haplotype Homozygosity (XP-EHH) method estimates the length of extended haplotypes and evaluates differences between two populations. It has previously been used to detect breed specific signatures of selection in domestic species [15–17]. The XP-EHH analysis effectively detects signals of differentiation across breeds and it has previously been used to detect putative loci affecting height in Shetland ponies [18]. Genomic regions associated with selection for racing performance were identified by a scan for selective sweeps using whole-genome sequencing data from Thoroughbreds and the native Jeju breed [19].

The aim of the present study was to detect genomic regions under selection in Swedish Warmblood horses (SWB). In line with previous studies where comparisons between breeds were used to highlight signatures of selection [13, 19], we performed a genome scan for signatures of selection in SWB horses and Exmoor ponies. The comparison with Exmoor ponies is justified as the Exmoor Pony Society primarily aims to protect the heritage of the breed by preserving its genetic diversity [20], and therefore selection for sport performance traits is not practiced [21]. A recent study showed that the Exmoor pony did not exhibit any common homozygous region with breeds intensively selected for sport performance, which supports its suitability for the purpose of this study [12]. Based on the hypothesis that performance-oriented selective breeding increased genomic homozygosity in specific regions in SWB horses, we used two different approaches: 1) analysis of ROH detected in SWB and Exmoor ponies, and 2) two population differentiation tests, F_st_ and XP-EHH analysis, comparing SWB and Exmoor ponies.

## Results

### Genotyping, quality control, and inbreeding coefficients

The total genotyping call rate was 0.99 in SWB (*n* = 380), and 0.97 in Exmoor ponies (*n* = 274). The LD measured by the squared correlation coefficient (r^2^) showed significant mean differences (*p* < 0.005) between the two populations. In SWB horses, the average r^2^ was 0.56 at 5 kb distance, compared to 0.60 in the Exmoor ponies. The estimated inbreeding coefficient based on loss of heterozygosity (f_i_) in the 380 SWB ranged from − 0.134 to 0.098 with an average value of 0.006. The f_i_ values in the 274 Exmoor ponies ranged from − 0.378 to 0.530, with an average of 0.170. Overall, 129 Exmoor ponies and six SWB horses were excluded from further analyses as their f_i_ exceeded the threshold of 5% set in this study.

### ROH as genomic signatures of selection

**Fig. 1:**
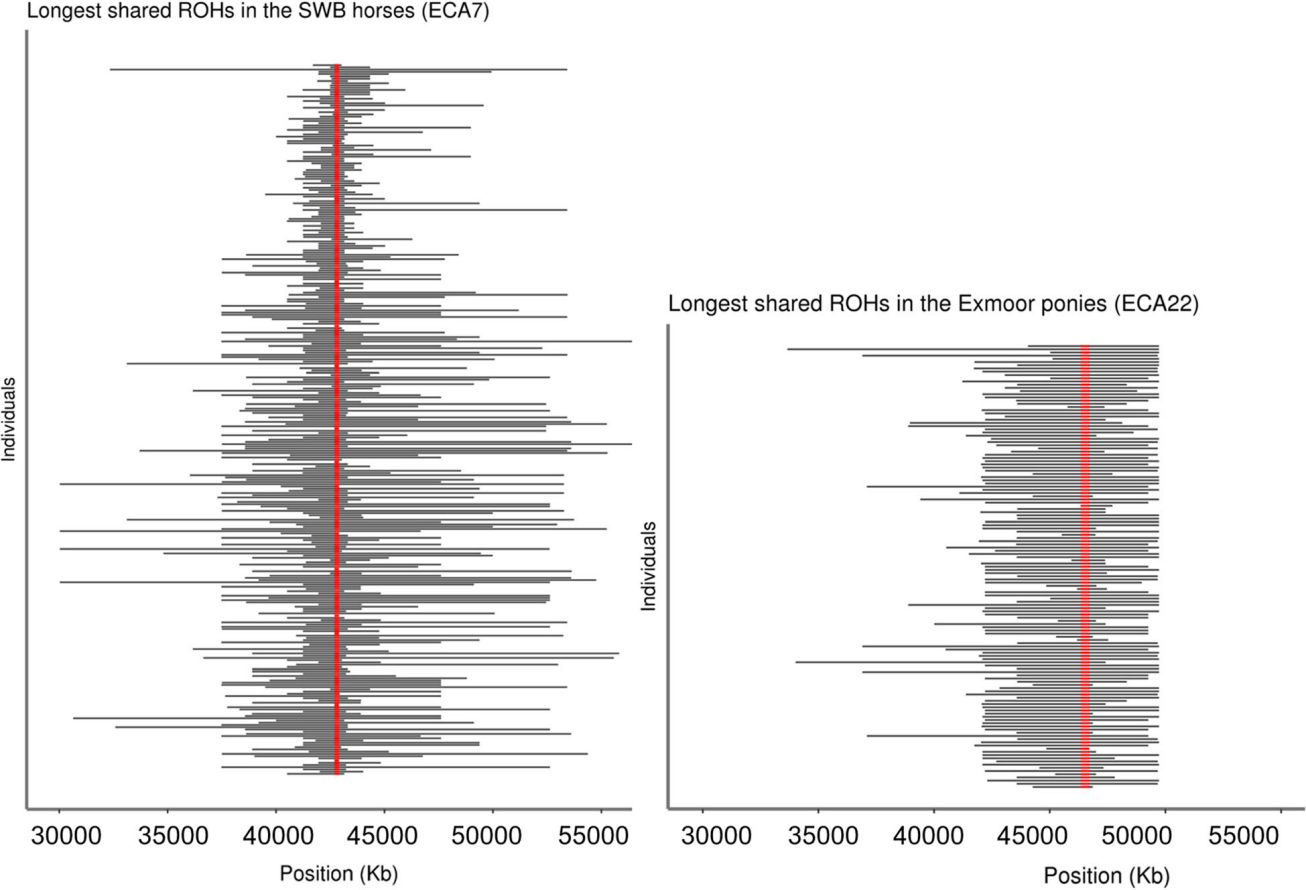
Interval of the longest shared homozygosity in SWB horses and Exmoor ponies. ROH length per each individual is indicated as horizontal black line. The red line indicates the shared interval among over 85% of the population

**Fig. 2:**
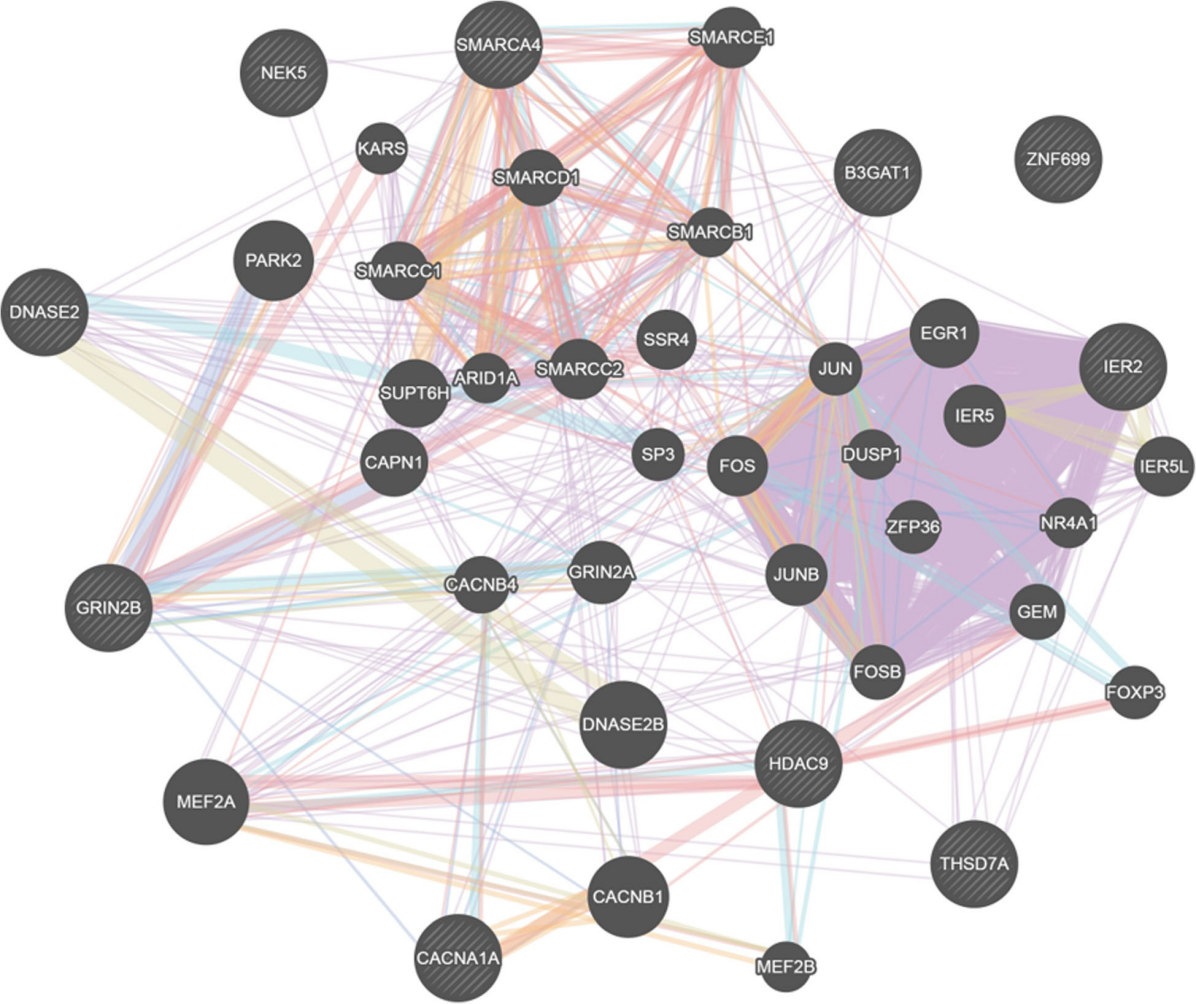
GeneMANIA representation of the genes found in the shared ROH in the SWB horses. The ten annotated genes are represented as stripped grey circles. Physical interactions are displayed as red lines, co-expressions as violet lines, predicted related genes as orange lines, shared pathways as light blues lines, co-localisations as blue lines and genetic interaction as green lines. The three most related genes with the potentially under selection ones are shown as plain circle

The average number of both short (< 125 kb), medium (125–500 kb) and long (> 500 kb) ROH per individual was higher in the Exmoor ponies than in the SWB horses (*p* < 0.0001); short ROH were five times more abundant in the Exmoor ponies than in SWB horses (Table 1). The average number of short ROH per animal was twice as high when including all the 274 Exmoor ponies that passed the QC, compared to when only including the 145 ponies with a f_i_ < 5% in the analysis, which supported the use of the f_i_ threshold. Short and medium ROH were distributed equally along the genome in both SWB and Exmoor ponies. Long ROH were the rarest ones as shown in Table 1; nevertheless, such ROH covered, on average 286 Mb in SWB horses and 1182 Mb in Exmoor ponies. While long ROH in Exmoor ponies were spread across the genome, long ROH in the SWB population were found in a few chromosomes only. The same pattern was discovered when filtering long homozygous regions shared by at least 85% of SWB horses and Exmoor ponies, respectively. The 65 long shared ROH detected among SWB horses contained overlapping homozygous segments in five chromosomes: ECA4, ECA6, ECA7, ECA10 and ECA17 (Additional file 1: Figure S1a). In Exmoor ponies, 398 long shared homozygous segments were instead distributed over 24 out of 31 (77%) autosomal chromosomes (Additional file 1: Figure S1b). The longest shared homozygous segment within ROH in SWB horses was 0.28 Mb and was located on ECA7:42,688,962-42,905,689, whereas in Exmoor ponies the longest shared homozygous region was 0.37 Mb on ECA22:46,333,459-46,708,156 (Fig. 1). The exact overlap in a ROH in ECA7 (7:49,160,767:49,212,921) coincided with a known QTL regions for body size (height at withers) [22]. The homozygous long ROH segments, shared by more than 85% of the studied Exmoor ponies, harboured 265 genes (Additional file 3: Table S1). In SWB, only 21 genes were located in the overlapping homozygous segments detected by long ROH analysis. Eighteen of them were annotated in the reference genome EquCab2 release 94 where ten had an annotated function, and eight were novel genes. The gene position, name and percentage of horses sharing an exact overlap in a ROH are listed in Table 2. All but one of the ten annotated genes located in shared ROH in SWB were included in a network based on co-expression, physical interactions or shared protein domains (Fig. 2). From the set of candidate genes five significantly overrepresented biological processes were found; actin cytoskeleton reorganization (GO:0031532), cellular macromolecule catabolic process (GO:0044265), apoptotic process (GO:0006915), glycoprotein metabolic process (GO:0009100) and synaptic signalling (GO:0099536) (Additional file 4: Table S2).

**Table 1:** Descriptive summary of ROH identified following three procedures (short, medium and long ROH detection) per breed: SWB horses and Exmoor ponies

Breed	Short ROH			Medium ROH			Long ROH		
	Total N.^a^	Mean N.^b^	Mean L. (Kb)^c^	Total N.	Mean N.	Mean L. (Kb)	Total N.	Mean N.	Mean L. (Kb)
SWB (*N* = 374)	257,419	688	76	374,665	1001	445	184,846	494	580
Exmoor pony (*N* = 145)	515,207	3553	82	171,149	1180	260	96,765	667	1773

^a^Total N.: Total number of ROH detected in the population

^b^Mean N.: average number of ROH per individual calculated as the Total N. divided by the number of individuals: 374 in the SWB horses and 145 in the Exmoor Ponies

^c^Mean L.: average length of ROH expressed in Kb

**Table 2:** Genes found in overlapping homozygous segments found within long ROH in over 85% of the SWB horses

Exact overlap in a ROH^a^	Gene symbol	Gene name	% of SWB
4:44,468,835:44,656,577	*THSD7A*	Thrombospondin type 1 domain containing 7A	86.0%
4:50,825,457:50,825,457	*HDAC9*	Histone deacetylase 9	86.6%
6:41,324,520:41,661,196	*GRIN2B*	Glutamate ionotropic receptor NMDA type subunit 2B	88.0%
7:42,688,962:42,905,689	*B3GAT1*	Beta-1,3-glucuronyltransferase 1	85.0%
	*ENSECAG00000009503*	Novel pseudogene	85.0%
	*ENSECAG00000003683*	Novel gene -	85.0%
7:45,280,925:45,280,925	*IER2*	Immediate early response 2	91.0%
7:44,827,476:45,014,430	*CACNA1A*	Calcium voltage-gated channel subunit alpha1 A	93.6%
7:45,525,088:45,525,088	*DNASE2*	Deoxy-ribonuclease 2, lysosomal	94.0%
7:47,244,532:47,445,238	*ENSECAG00000010696*	Novel gene	90.4%
	*ENSECAG00000012790*	Novel gene	90.4%
	*ENSECAG00000004301*	Novel gene	91.2%
7:48,419,051:48,419,051	*ENSECAG00000020829*	Novel gene	91.2%
7:49,160,767:49,212,921	*SMARCA4*	SWI/SNF related, actin dependent regulator of chromatin	86.0%
7:50,250,257:50,286,784	*ENSECAG00000015373*	Novel gene	85.8%
	*ZNF699*	Zinc finger protein 699	89.0%
7:51,068,391:51,107,956	*ENSECAG00000002451*	Novel gene	91.4%
7:51,514,450:51,560,980	*ENSECAG00000002839*	Novel gene	91.2%
10:29,104,190:29,163,955	*ENSECAG00000003641*	Novel pseudogene	88.0%
	*ENSECAG00000025061*	Novel pseudogene	88.0%
17:19,157,873:19,157,873	*NEK5*	NIMA related kinase 5	85.1%

^a^Homozygous segments shared by over 85% of SWB horses within long ROH

### Breed differentiation tests

**Fig. 3:**
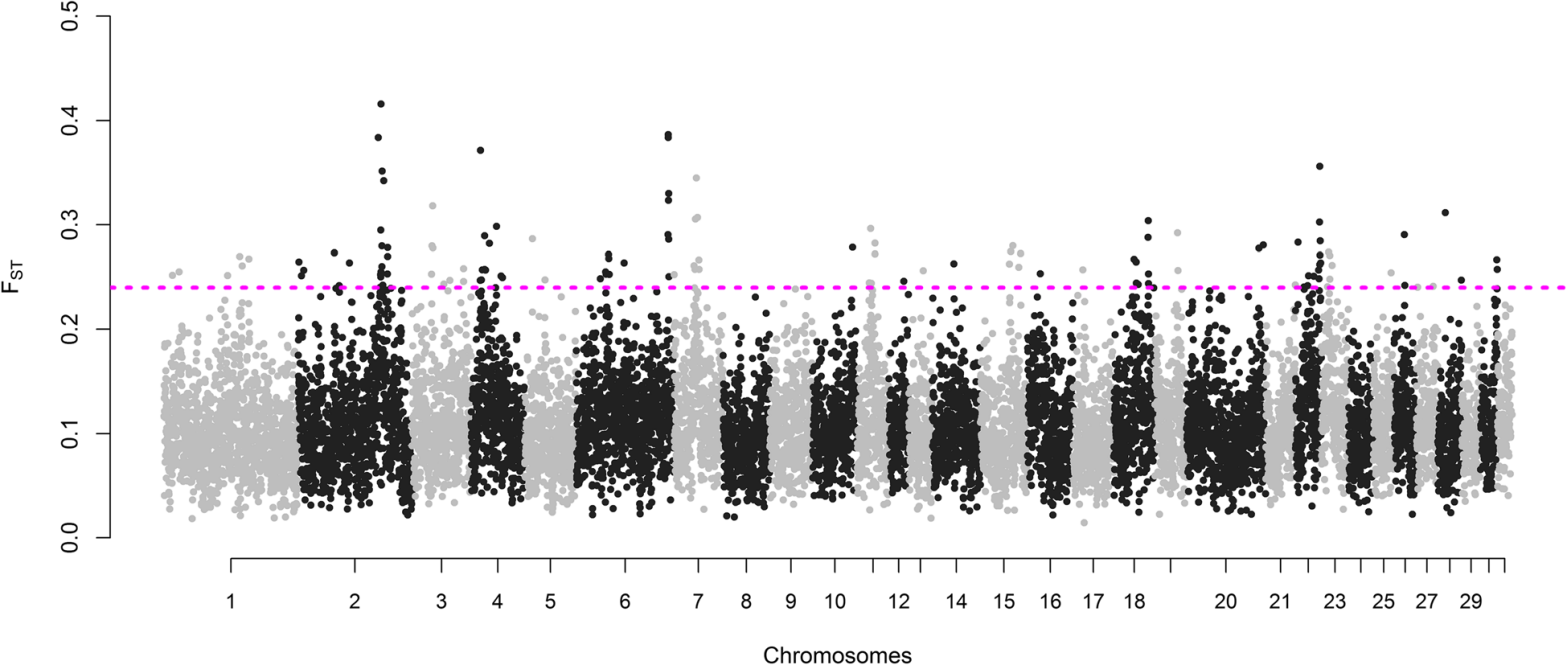
Genomic distribution of averaged F_st_ values in 500-kb windows plotted relative to their physical position within each autosomal chromosome. The cut-off to call a SNP as significant was defined as the highest 1% of the empirical distribution and is represented by the SNPs above the dotted pink line (F_st_ ≥ 0.24)

The average F_st_ for all windows between SWB and Exmoor ponies was equal to 0.11 with a standard deviation of 0.04. The distribution of F_st_ estimates per SNP is shown in Additional file 2: Figure S2. Average F_st_ per window for a total of 126 windows exceeded the significance threshold, corresponding to an averaged F_st_ value ≥0.24 (Fig. 3). The top 10 windows with highest F_st_ were located on ECA2, ECA4, ECA6, ECA7 and ECA22.

**Fig. 4:**
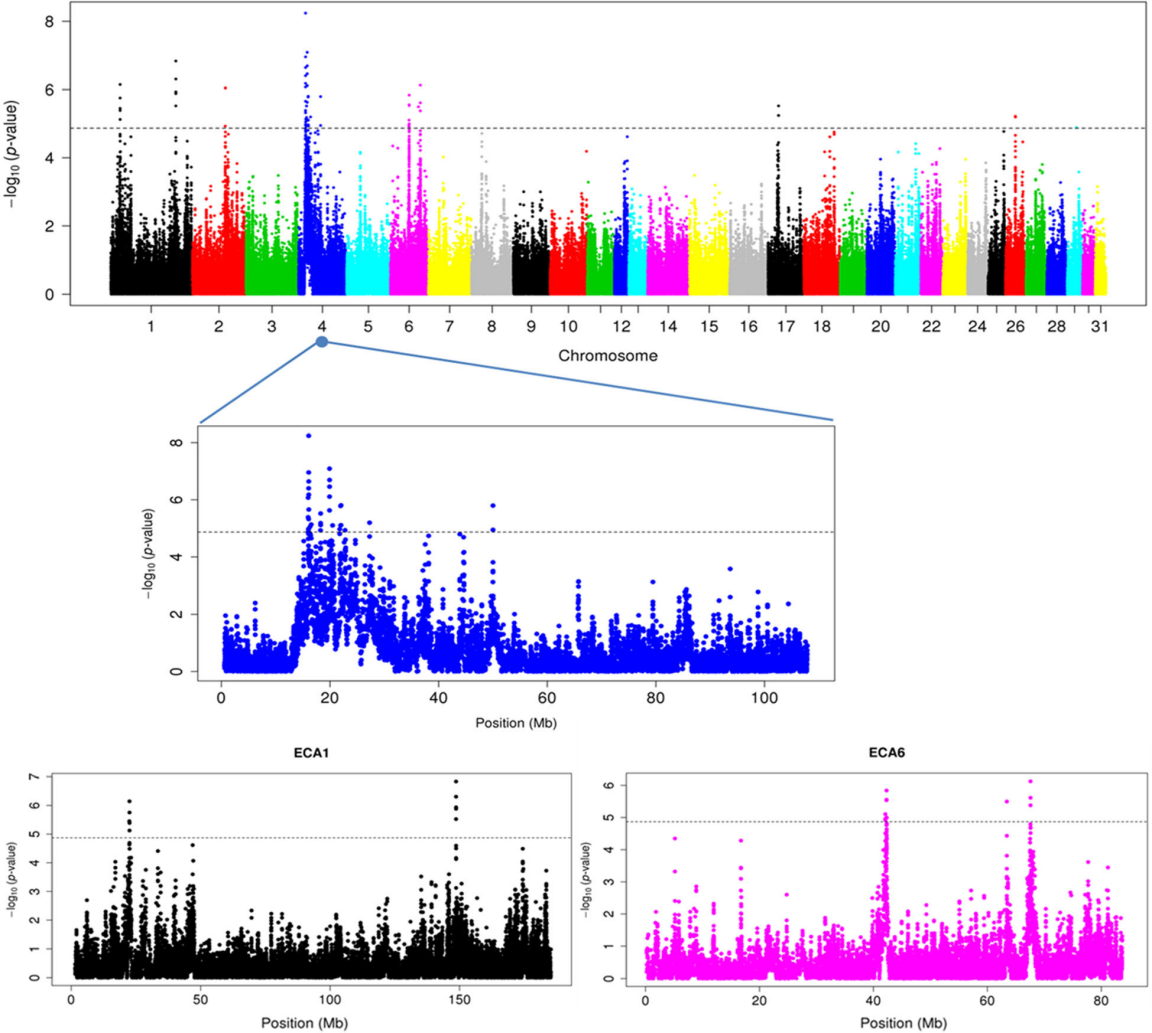
Genome scan of potential regions under selection detected by the cross populations expended haplotype homozygosity (XP-EHH) test. The log10(*p*-value) is plotted for each SNP per chromosome (top panel). The chromosomes ECA1, ECA4 and ECA6 contain significant SNPs, are zoomed in and displayed in the middle and lower panels

Genomic regions with an XP-EHH value above 4.34 and thus potentially under positive selection in SWB horses were detected on ECA1, ECA2, ECA4, ECA6, ECA17 and ECA26. The complete list of markers underlining potential signatures of selection is presented in Additional file 5: Table S3. Eight signatures of selection located on ECA1, ECA4, and ECA6 in SWB horses remained significant after correcting for false discovery rate (Fig. 4).

**Fig. 5:**
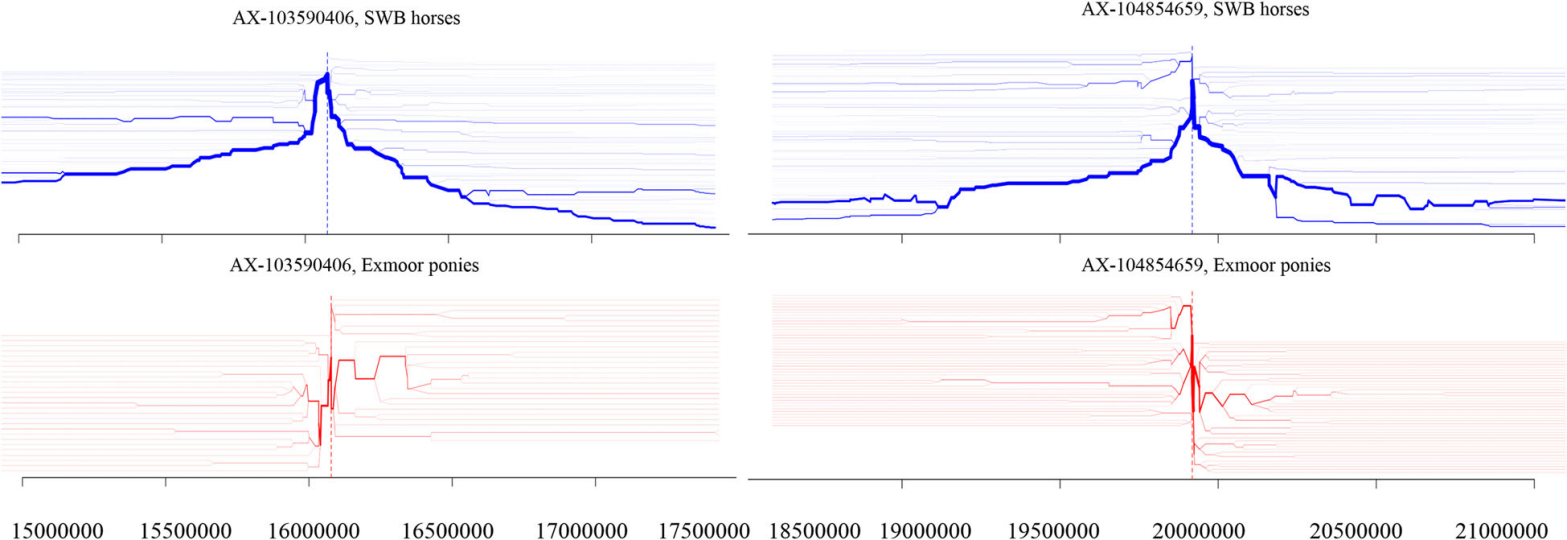
Bifurcation diagram for the two focal SNPs on chromosome 4: position 16,077,767 and 19,918,093. The two core SNPs were defined as the two with the highest XP-EHH result from the cross populations extended haplotype homozygosity test (XP-EHH = 5.76 and XP-EHH = 5.36). The two plots represent the LD breakdown at increasing distances from the core allele at the selected focal SNP in the SWB horses (on top and represented as blue lines) and in the Exmoor ponies (at the bottom and represented as red lines)

The breakdown of linkage disequilibrium from the two SNPs with the highest XP-EHH estimate (XP-EHH = 5.82 for ECA4:16,077,767 and XP-EHH = 5.36 for ECA4: 19,918,093) is shown in Fig. 5. No position with an XP-EHH value lower than − 4.34 was found in Exmoor ponies, indicating a lack of recent selection. Four genomic regions were detected by both F_st_ and XP-EHH population differentiation tests. The genes located within those regions were considered as potentially under selection in SWB horses (Table 3).

**Table 3:** Genomic regions detected by both Fst and XP-EHH which were further considered as signs of selection

Chromosome	Position (bp)			Average XPEHH^a^	Average F_st_^a^	Genes
1	148,660,078	:	149,160,078	4.67	0.26	*FSIP1, THBS1*
4	15,977,755	:	16,477,755	5.82	0.37	*ADCY1, IGFBP1, IGFBP3*
4	19,913,037	:	20,413,037	5.09	0.26	*ZPBP, SPATA48, IKZF1, FIGNL1, DDC, GRB10*
6	42,096,241	:	42,596,241	4.81	0.25	*GUCY2C, WBP11, SMCO3, C6H12orf60, ART4, MGP, ERP27, ARHGDIB, PDE6H, RERG*

^a^XP-EHH and F_st_ averaged over 500-kb windows

The region on ECA 6 (6:42,096,241:42,596,241) overlapped with a QTL associated with body conformation in horses [23]. Six overrepresented biological processes were found from the list of potentially-selected genes; cellular response to stimulus (GO:0051716), ribonucleotide biosynthetic process (GO:0009260), signal transduction (GO:0007165), regulation of cell communication (GO:0010646), peptidyl-arginine modification (GO:0018195) and mRNA cis splicing, via spliceosome (GO:0045292) (Additional file 6: Table S4).

## Discussion

### Genomic traces of breed history

Selection in SWB horses for equestrian sport performance traits was highly intensified during the last decades. In 1973, a field test for young horses was introduced in the SWB breeding program to allow for a more accurate selection of the breeding animals and increased genetic gain [24]. The overlapping homozygous segments shared in most of SWB horses agree with the intensified selective breeding program applied by the SWB studbook in the last 40 years. We believe this is an indication that shared homozygous segments in SWB horses can be a result of recent selection for performance, rather than inbreeding. This is supported by the estimated inbreeding coefficient (f_i_) that was below 5% in all but six SWB individuals in our study and is likely to be a result of a semi-open studbook allowing inflow of genetic material from other warmblood populations. In contrast, ROH of all lengths were evenly spread across the genome of Exmoor ponies. Short ROH were as much as five times more frequent in Exmoor ponies than in SWB horses, indicating historical inbreeding in line with the Exmoor’s known demography. The Exmoor pony breed is listed as “endangered” by the Rare Breeds Survival Trust [25], and thus founder effect and genetic drift were expected in the breed. In agreement with such assumption, several Exmoor ponies showed high f_i_, which indicates significant loss of heterozygosity. Therefore, we applied inbreeding correction to distinguish between homozygosity due to relatedness rather than selection. Our results support that long shared ROH may have different origins due to different SWB horse and Exmoor pony population histories. In SWB horses, long ROH may originate from recent intensive selection for sport traits, whereas in Exmoor ponies long ROH may be the result of past bottlenecks. However, the difficulty to clearly define the origin of reduction in genetic diversity in small populations has been pointed out as they are vulnerable to genetic drift [26, 27]. Therefore, we did not further examine the results retrieved from ROH in the case of Exmoor ponies, but used the results as a comparison to the SWB.

In the current study, we performed a correction for linkage disequilibrium (LD) which has not been common practice for less dense SNP data for the ROH analysis. The LD measured by the squared correlation coefficient (r^2^) was significantly higher in the Exmoor ponies if compared to SWB horses, most likely because of breed history. Therefore, LD pruning was performed to reduce strong LD between markers originating from population history rather than from positive selection [28–30].

### Signatures of selection based on ROH in SWB horses

Performance traits are known to be complex traits caused by mutations in many genes and regulatory elements, generally connected in aggregated networks. Thus, it is not surprising that several of the genes indicated to be under selection in SWB horses share pathways, co-expression, co-localisation, and interact physically or genetically.

Selection for muscle strength and function is indeed a favoured trait in the performing horse, and we found several genomic regions comprising genes involved in this. An especially interesting example is the Histone deacetylase 9 (*HDAC9*) with a known regulatory function in neuronal electrical activity of excitable skeletal muscle cells [31]. Two of the ten annotated genes detected in long ROH share biological functions in synaptic signalling (GO:0099536); the Glutamate Ionotropic Receptor NMDA Type Subunit 2B gene (*GRIN2B*) and the Calcium Voltage-gated Channel Subunit 1 gene (*CACNA1A*). Both genes are involved in response to pain, synaptic transmission and receptor clustering. The protein encoded by *GRIN2B* is a NMDA receptor channel subunit critical for neuronal communication [32]. The *GRIN2B* gene has been pointed out as potentially important for performance in Icelandic horses and Coldblooded trotters [33, 34] and as a target of selection in French Trotters and Gidran horses [12]. Out of the 21 genes found in shared long ROH of SWB horses, *GRIN2B* was the only one found also in shared long ROH in Exmoor ponies. We therefore believe that this gene may have been under selection prior to horse breed formation. The gene *CACNA1A* encodes the protein C_av_ involved in muscle contraction, hormone and neurotransmitter release [35]. Behaviour analyses in mice suggested that C_av_ contributes to pain perception [36]. In humans loss-of-function mutations in the *CACNA1A* were implicated in neurological and psychiatric diseases [37]. Our ROH analysis also detected a region comprising the *Beta-1,3-glucuronyltransferase 1 (B3GAT1)* which has been associated with learning abilities in domesticated rats [38].

Genes involved in neurological control and signalling pathways were likewise found in shared ROH among Hanoverian sport horses [13]. The SWB breed is genetically connected with the Hanoverian breed, as well as with other European sport horse breeds [39]. This indicates that some of our presented findings are also applicable in other warmblood breeds used for sport and that cognitive reactions and functions may be important targets of selection in Warmblood breeds. This latter result agrees with previous studies where differences in temperament between breeds have been shown [40].

### XP-EHH and F_st_ tests indicate recent selection in SWB horses

The average F_st_ of 0.11 between the two breeds was similar to the previously estimated average F_st_ of 0.10 between 37 horse populations across the globe [29], indicating that a comparison between SWB and Exmoor ponies is reasonable. In this study, we investigated signatures of selection for performance traits in SWB horses by comparing them with a breed not selected for sport performances. We could confirm that even the worst-performing SWB horse was genetically superior in equestrian sports to the best Exmoor pony, thus excluding any potential confounding effects in our study. Although the comparison with an ancestral breed of SWB horses would have been desirable, none of the remaining native Swedish breeds can be claimed as the ancestor of SWB horses. Further comparison with other warmblood horse populations could also verify the validity of this study.

The XP-EHH analysis support our hypothesis that recent selection took place in SWB horses, but not in Exmoor ponies, as significant regions were only found in the former. Since the Exmoor pony genome presents widespread homozygous regions, we probably failed to identify all regions under selection in the SWB by comparing the two breeds. However, we believe that this comparison has considerably reduced the risk of finding false positives. Concordance across statistics is generally used to support putative sweeps, this is the reason why we only further analysed regions detected by both population differentiation tests [17, 40, 41]. Selection for eight genes involved in cellular response to stimulus (GO:0051716) were found from the composite results of the F_st_ and XP-EHH tests. As an example, the *Adenylate Cyclase 1* (*ADCY1)* gene encodes adenylyl cyclase (AC) primarily expressed in the brain, influences neuroplasticity, as long-term potentiation, depression and memory formation [42]. In mice, *ADCY1* and *ADCY8* play an important role in the formation and maintenance of fear memory, dopaminergic responses, and behavioural sensitisation [43, 44]. Additionally, we detected the *Guanylate Cyclase 2C (GUCY2C)* which is associated with human attention deficiency and hyperactive behaviour [45], along with the *Rho GDP Dissociation Inhibitor Beta (ARHGDIB)*, and RAS *Like Estrogen Regulated Growth Inhibitor (RERG)* genes [46, 47]. These four genes (*ADCY1, GUCY2C, ARHGDIB, and RERG)* are all involved in G-protein coupled receptor, and GTPase mediated signal transduction, common in the central nervous system, suggesting an important role in learning and reactivity in the performing horse.

Two other genes, represented in the cellular response to stimulus term, shared biological function related to signal transduction (GO:0007165) and regulation of cell communication (GO:0010646): The *Insulin like growth factor binding protein* 1 (*IGFBP1)* and the *Insulin like growth factor binding protein 3 (IGFBP3)*. These were also detected in studies of selection signatures in German Warmblood breeds [11] and in French Trotters [12]. Both are members of the insulin-like growth factor binding protein (IGFBP) family that binds IGF-I and -II and regulates somatic growth with an important function in muscle growth distribution. The importance of growth traits was further supported by two detected regions overlapping with known QTLs related to conformation and morphology traits (body size and cannon bone circumference) [22, 23].

In agreements with other studies, the region on ECA4:19,913,037-20,413,037, in our study, seems to be under selection in warmblood horses [11, 40]. This region contains several genes, for example *Spermatogenesis associated 48 (SPATA48*) and *Zona pellucida binding protein* (*ZPBP)*, which play a role in equine fertility [11, 40]. Also, the most significant SNP in this region in our XP-EHH test was located within the *ZPBP* gene (Fig. 5)*. SPATA48* also promotes osteoblast differentiation, which might have an important function in sport horses [48, 49]. In our study the overrepresentation test showed as significant gene the *Fidgetin Like 1 (FIGNL1)* which has likewise a known function in osteoblast differentiation [50]. This gene was also found in the top ten enriched pathways from the iHS test in four German warmblood horse breeds [11]. In two other regions on ECA 4 and ECA7, our ROH analysis detected the *Thrombospondin, type I (THSD7A)* and the *Deoxy-ribonuclease 2, lysosomal (DNASE2)*, respectively, which are both involved in bone metabolism [51, 52]. These findings may indicate a link between fertility and bone metabolism.

To further validate our findings, sport performing horses should be re-sequenced to find candidate causative mutations, and functional studies are needed to confirm biological effects of the mutations.

## Conclusions

We conclude that genes associated with behavioural, physical abilities, and fertility appear to be targets of selection in the SWB horse breed. Our analysis of SWB horses reveal putative signatures of selection in genomic regions containing genes primarily involved in nervous system functionality, as well as muscle contraction and development. As expected, due to the complex nature of sport horse performance, many of the genes under selection in SWB interact with each other in complex biological networks. In line with this, our study reveals that selection for sport performance has likely occurred in numerous genomic regions. Our work unveils the effects of selection in sport horses primarily used for riding and highlights how selection has shaped the genome of Swedish Warmblood horses, a representative breed for modern sport horses.

## Methods

### Sample collection

The study included 380 Swedish Warmblood horses born in 2010–2011. The horses (182 males and 198 females) were assessed at young horse evaluation tests at the age of three [53] and descended from 145 sires with 1–11 offspring each, and 372 mares with 1–2 offspring each. The selected horses were either: *1)* horses with high scores for show jumping but lower ones for gaits (*n* = 48), *2)* horses with high scores for gaits but lower ones for show jumping (*n* = 48), *3)* horses with high scores for both show jumping and gaits (*n* = 143), and *4)* horses with low scores for both show jumping and gaits (*n* = 141). Genotype information (670 k SNP array data) of 280 Exmoor ponies was retrieved for comparison from a previous publication, which includes details about the ponies’ selection procedure for genotyping [21].

### DNA isolation

DNA was prepared from 20 hair roots, cut into 5% Chelex 100 Resin (Bio-Rad Laboratories, Hercules, CA, US), and 1.4 mg/ml Proteinase K (Merck KgaA, Darmstadt, Germany) in a total volume of 200 μl. The samples were incubated at 56 °C, 1500 rpm for 2 h, followed by heat inactivation of Proteinase K at 96 °C for 10 min. DNA concentration was normalised, and the DNA was re-suspended in lowTE (1 mM Tris, 0.1 mM EDTA) at a concentration of 10 ng/μl.

### Genotyping, quality control and inbreeding coefficients

All samples were genotyped using the 670 K Affymetrix® Axiom® Equine Genotyping Array (Thermo Fisher Scientific, Santa Clara, CA, USA) [54]. Individual inbreeding coefficients (f_i_) were estimated from loss of heterozygosity using the PLINK *–het* command and horses with an f_i_ higher than 0.05 were excluded for further analyses. Quality Control (QC) was performed separately for each breed on the 31 autosomal chromosomes. The exclusion of poorly genotyped and faulty data was performed using PLINK v1.90 [55] based on the following criteria: minor allele frequency (MAF) (< 0.01), missing genotype per single SNP (GENO) (> 0.10), missing genotype per individual (> 0.10) and Hardy-Weinberg equilibrium (HWE) (*p* < 0.0001). A linkage disequilibrium pruning was applied for the ROH analyses. SNPs in linkage disequilibrium (LD) were excluded if the LD between each pair of SNPs was greater than 0.5 (r^2^ > 0.5) in a window size of 50 SNPs moving 5 SNPs per window. In total 249,395 SNPs were used for ROH analysis.

### Definition of ROH

ROH were detected in SWB and Exmoor separately, using a sliding-windows approach through the *homozyg* command in PLINK v1.90. As different signatures of selection are expected when using various ROH definitions, we classified ROH as short (< 125 kb), medium (125 kb to 500 kb) and long (> 500 kb). The three-length classes were defined by the following criteria: minimum number of SNPs, minimum SNP density (SNP/kb), maximum gap between two SNPs (bp) and the minimum length to define a ROH (kb). The sliding window was defined by using the options *homozyg-window-snp*, *homozyg-window-missing* and *homozyg-window-het* in PLINK v 1.90 (Table 4). The last two options were only applied when detecting long stretches of homozygosity, thus allowing for one heterozygous and one missing SNP [56].

**Table 4:** Procedures used to define short, medium and long ROH and the sliding windows’ parameters after QC

Type	Criteria to define ROH				Sliding window			
	Num. SNPs	Density (SNP/kb)	Max gap (bp)	Length (kb)	Size (kb)	Het.^a^ SNPs	Miss.^b^ SNPs	
Short	3	1/100	5000	50–125	5	0	0	
Medium	10	1/100	5000	125–500	12	0	0	
Long	30	1/100	5000	> 500	50	1	1	

^a^Het: Number of heterozygous SNPs allowed in the sliding window

^b^Miss: Number of missing SNPs allowed in the sliding window

The differences in the number of ROH in each ROH length-class between the two breeds were tested by a one-way analysis of variance in R (v3.4.0) [57]. Proportional differences of ROH between breeds and uniformity of ROH over each respective chromosome were tested with the Chi square test (χ^2^) for proportions and goodness of fit in R.

### ROH as genomic signatures of selection within breed

A custom-made script in R was used to filter homozygous regions within long ROH shared by more than 85% of the studied individuals within breed. The use of 85% as threshold was chosen to identify regions containing fundamental loci for breed-type shared by most SWB horses, regardless of which equestrian sport discipline they were bred for, and thus enable detection of important loci for general sport performance. The EqCab2 genomic coordinates of these regions were used to retrieve candidate gene lists and annotations from the Biomart web interface in Ensembl release 94 [58]. Genetic co-expression, physical interactions or shared protein domains, were visualised by GeneMANIA in Cytoscape with human genes as reference [59]. Additionally, genes were compared with QTL regions previously identified and present in the Horse QTL database [60]. Statistical overrepresentation test of biological processes (GO terms) of candidate genes was conducted using PANTHER 14.0 (http://pantherdb. org/) [61]. The level of significance for the overrepresented biological processes was set as *p* < 0.05.

### Population differentiation tests

The genetic differentiation between SWB and Exmoor ponies was verified by the fixation index (F_st_) as defined by Nei (1987) [62]. To identify highly differentiated regions, we divided the genome into non-overlapping 500 kb windows. A F_st_ value was calculated for each SNP and the values were then averaged over the SNPs located in each window. Windows located at the extreme 1% of the empirical distribution of F_st_ values were considered as candidate regions [63].

For analysis of population differentiation at haplotype level, haplotypes were phased using Shape-it software [64] and filtered using REHH Package in R [65], resulting in 503,829 SNPs. We then used XP-EHH statistics to identify regions displaying significantly higher, or lower, extended haplotype homozygosity in one population compared to the other [14]. A position was considered under selection if the XP-EHH in two population-pairwise comparisons was above 4.34 or below − 4.34 as suggested by [14]. To minimise the number of false positives and to account for large variation, selection signatures were averaged over windows. Only regions containing SNPs falling into the upper 99th percentile, defined as 500 kb windows, and containing at least three SNPs were considered as putative signatures of selection. A haplotype bifurcation diagram around a core marker was used to visualise signatures of selection indicated from the XP-EHH.

Furthermore, genes located in genomic regions containing significant SNPs based on both F_st_ and XP-EHH analyses were retrieved using Biomart, as previously described, and potential overlaps with QTLs, present in the Horse QTL database [66], were considered. Statistical overrepresentation test of biological processes (GO terms) of the candidate genes was conducted using PANTHER 14.0 as described above.

## Supplementary information

**Additional file 1: Fig. S1.** Incidence of each single nucleotide polymorphism (SNP) in ROH in the SWB horses and Exmoor ponies. Genomic positions highlighted in green represent SNPs in a homozygous segment shared in over 85% of the SWB horses (a) and Exmoor ponies (b). The blue line shows the threshold for SNPs present in more than 80% of the horses and the red line shows the average value (22%) of SNP incidence in homozygous segment in SWB horses and (51%) in Exmoor ponies.

**Additional file 2: Fig. S2.** Distribution in frequency class of Fixation index (F_st_) between SWB horses and Exmoor ponies.

**Additional file 3: Table S1.** Genes located within consensus homozygous segments in Exmoor ponies.

**Additional file 4: Table S2.** Details on the five overrepresented biological processes and the genes involved in each biological term from the ROH analysis.

**Additional file 5: Table S3.** SNP markers found to be under potential selection when analysed by the XPEHH test before FDR control.

**Additional file 6: Table S4.** Details on the six overrepresented biological processes and the genes involved in each biological term from the overlapping regions from the XPEHH and F_st_ tests.

## Data Availability

The datasets in the current study was generated and analysed in collaboration with the Swedish Warmblood Association and has a commercial value for them. The SWB horse data is therefore available from the corresponding author on reasonable request. The Exmoor pony genotypes are publicly available in our previous study [21]. All relevant data from the Exmoor ponies are available via Figshare (DOI: 10.6084/m9.figshare.3145759).

## Abbreviations

AC: Adenylyl cyclase
ADCY1: Adenylate Cyclase 1
ADCY8: Adenylate Cyclase 8
ARHGDIB: Rho GDP Dissociation Inhibitor Beta
B3GAT1: Beta-1,3-glucuronyltransferase 1
CACNA1A: Calcium Voltage-gated Channel Subunit
C_av_: Voltage-dependent calcium channels
DNASE2: Deoxy-ribonuclease 2 lysosomal
EBV: Estimated breeding value
f_i_: Individual inbreeding coefficient
GRIN2B: Glutamate Ionotropic Receptor NMDA Type Subunit 2B
GTPase: Guanosine-5′-triphosphate hydrolase
GUCY2C: Guanylate Cyclase 2C
HDAC9: Histone deacetylase 9
HWE: Hardy-Weinberg equilibrium
iES: Integrated extended haplotype score
IGFBP1: Insulin like growth factor binding protein 1
IGFBP3: Insulin like growth factor binding protein 3
LD: Linkage disequilibrium
MAF: Minor allele frequency
QC: Quality control
RERG: RAS Like Estrogen Regulated Growth Inhibitor
ROH: Runs of homozygosity
SNP: Single nucleotide polymorphism
SPATA48: Spermatogenesis associated 48
SWB: Swedish Warmblood
THSD7A: Thrombospondin, type I
XP-EHH: Extended haplotype homozygosity
ZPBP: Zona pellucida binding protein
χ^2^: Chi square test
