# Supplementary figures and images for: Signatures of selection in the genome of Swedish warmblood horses selected for sport performance
Source: BMC Genomics. 2019 Sep 18;20:717. doi: 10.1186/s12864-019-6079-1 (PMC6751828; doi:10.1186/s12864-019-6079-1)

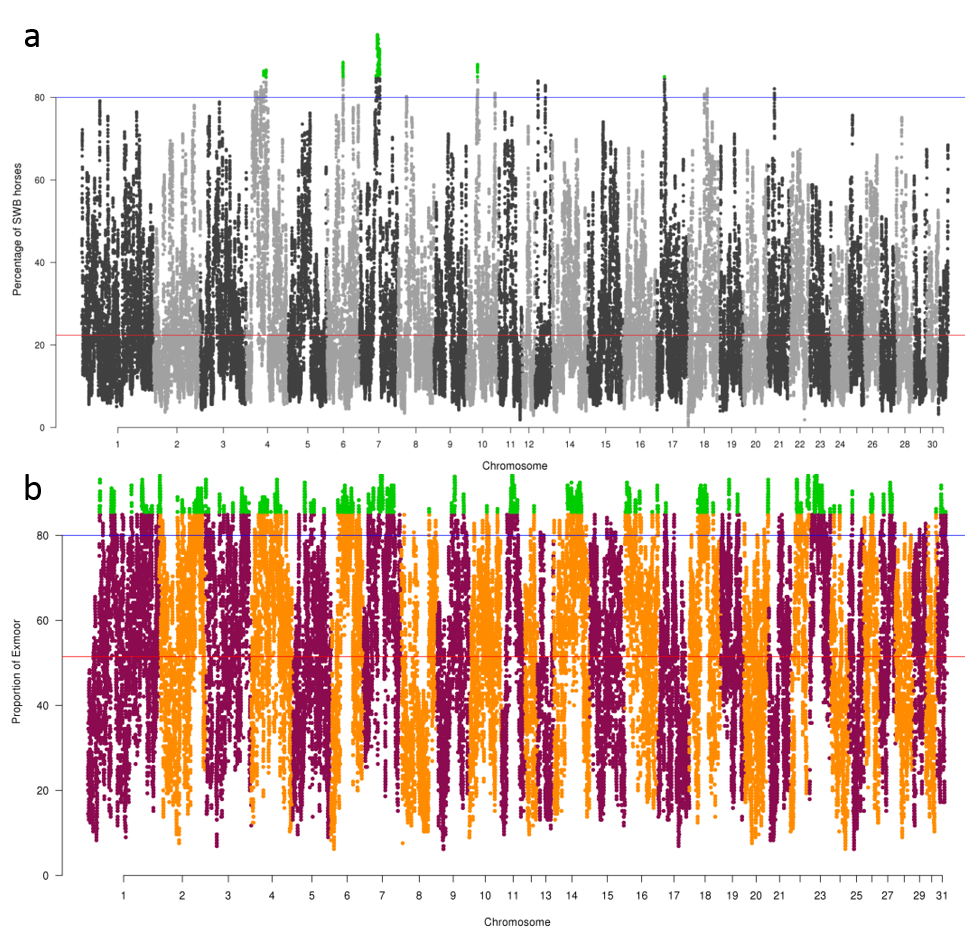

Supplement: Supplementary file 1 — Additional file 1: Fig. S1. Incidence of each single nucleotide polymorphism (SNP) in ROH in the SWB horses and Exmoor ponies. Genomic positions highlighted in green represent SNPs in a homozygous segment shared in over 85% of the SWB horses (a) and Exmoor ponies (b). The blue line shows the threshold for SNPs present in more than 80% of the horses and the red line shows the average value (22%) of SNP incidence in homozygous segment in SWB horses and (51%) in Exmoor ponies. [file 12864_2019_6079_MOESM1_ESM.png]

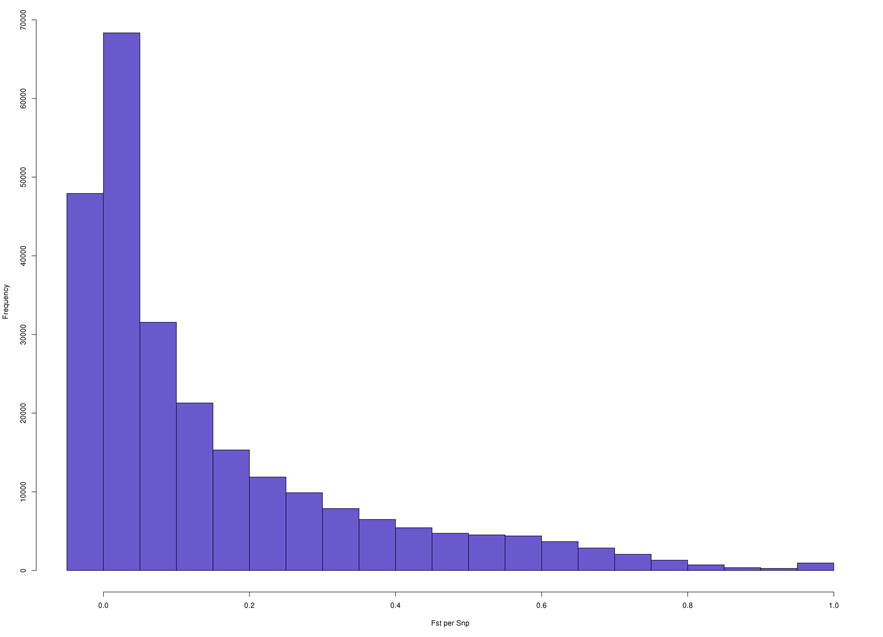

Supplement: Supplementary file 2 — Additional file 2: Fig. S2. Distribution in frequency class of Fixation index (Fst) between SWB horses and Exmoor ponies. [file 12864_2019_6079_MOESM2_ESM.png]
